# Magnetic-field induced rotation of magnetosome chains in silicified magnetotactic bacteria

**DOI:** 10.1038/s41598-018-25972-x

**Published:** 2018-05-16

**Authors:** Marine Blondeau, Yohan Guyodo, François Guyot, Christophe Gatel, Nicolas Menguy, Imène Chebbi, Bernard Haye, Mickaël Durand-Dubief, Edouard Alphandery, Roberta Brayner, Thibaud Coradin

**Affiliations:** 1 Sorbonne Université, CNRS, Collège de France, Laboratoire de Chimie de la Matière Condensée de Paris, 4 Place Jussieu, 75252 Paris, Cedex 05 France; 2 Sorbonne Université, Museum National d’Histoire Naturelle, CNRS, Institut de Recherche pour le Développement, Institut de Minéralogie, de Physique des Matériaux et de Cosmochimie, 75252 Paris, Cedex 05 France; 3 Interfaces, Traitements, Organisation et Dynamique des Systèmes (ITODYS), CNRS-UMR 7086, Université Paris 7 Denis Diderot, 15 rue Jean de Baïf, 75205 Paris, cedex 13 France; 4 Centre d’Élaboration de Matériaux et d’Etudes Structurales (CEMES), CNRS-UPR 8011, Université de Toulouse, 29 rue Jeanne Marvig, 31055 Toulouse, France; 5 Nanobactérie, Campus de l’Université d’Orsay, Batiment 201 rue Henri Becquerel, 91440 Bures-sur-Yvette, France; 6 Department of Biosciences and Nutrition, Karolinska Institutet, SE-141 83 Hudding, Sweden

## Abstract

Understanding the biological processes enabling magnetotactic bacteria to maintain oriented chains of magnetic iron-bearing nanoparticles called magnetosomes is a major challenge. The study aimed to constrain the role of an external applied magnetic field on the alignment of magnetosome chains in *Magnetospirillum magneticum* AMB-1 magnetotactic bacteria immobilized within a hydrated silica matrix. A deviation of the chain orientation was evidenced, without significant impact on cell viability, which was preserved after the field was turned-off. Transmission electron microscopy showed that the crystallographic orientation of the nanoparticles within the chains were preserved. Off-axis electron holography evidenced that the change in magnetosome orientation was accompanied by a shift from parallel to anti-parallel interactions between individual nanocrystals. The field-induced destructuration of the chain occurs according to two possible mechanisms: (i) each magnetosome responds individually and reorients in the magnetic field direction and/or (ii) short magnetosome chains deviate in the magnetic field direction. This work enlightens the strong dynamic character of the magnetosome assembly and widens the potentialities of magnetotactic bacteria in bionanotechnology.

## Introduction

Since their discovery more than 30 years ago, magnetotactic bacteria have received much attention in the fields of geological, chemical, physical and biological sciences^1–5^. However, the processes by which they synthesize iron oxide (or sulphide) magnetic nanoparticles and organize them into chains allowing the cell to orient itself along the geomagnetic field are still far from being fully understood^6–10^. It is well-admitted that production of the nanocrystals occurs in specialized organelles, called magnetosomes, formed by a membrane invagination at multiple sites in the cell. However, identifying the proteins controlling nucleation, size, shape and arrangement in chains of the nanocrystals, as well as the mechanisms of action of these proteins, remains a challenging task^11–15^.

Phenotypic divergences between different species were evidenced^16^. In some *Magnetospirillum* sp. strains, the chain is stabilized along the longitudinal axis of the cell by an organic filament, MamK protein, while the connection of magnetosomes to this structure is provided by a protein MamJ^17,18^. The deletion of *MamK* gene in *Magnetospirillum gryphiswaldense* MSR-1 leads to short chains separated by gaps devoid of magnetosomes, while the deletion of *MamJ* induces aggregation of magnetosomes within the cell with observable filaments not attached to the biogenic particles^19,20^. In *Magnetospirillum magneticum* AMB-1, additional MamJ-like and MamK-like proteins were identified that are involved in the dynamic of the filament and the magnetosome organization in chain^21–23^.

The capacity of the magnetosome chain to act as a compass is not only related to the spatial arrangement of nanocrystals, but also to their crystallographic and magnetic alignment. In some magnetotactic bacteria (e.g., *Magnetospirillum* sp.), the biogenic magnetite nanoparticles have a cubo-octahedral morphology with a magnetic easy axis [111] aligned along the chain axis^24,25^. The magnetization of a given single domain crystal is also oriented parallel with respect to the others in the chain thus forming a larger-scale magnetic dipole^26^. It could be expected that an external magnetic field present during the formation of magnetosomes, or once the chains are formed, could affect the organization of these biogenic structures. Few reports are available on the effect of an external magnetic field on the biomineralization process itself^27–30^. The interactions between already-formed magnetosomes and an external magnetic field have been so far mainly studied on free-moving bacteria^1,31,32^ or cell-extracted magnetosomes^33^. In one report, magnetotactic bacteria were immobilized in agarose gels, allowing for the observation a field-induced re-orientation of the chains without rotation of the whole bacterial cell^34^. Importantly the chain deviation was shown to be reversible when the field was turned off, suggesting that the assembly of proteins involved in the nanocrystal organization had a dynamic character.

These intriguing observations opened many additional questions such as the effect of chain re-orientation on cell viability as well as its impact on the crystallographic and magnetic orientation of the nanoparticles. Aiming to go further in the understanding of these processes, we have used here a mineral matrix, made of hydrated silica, as an encapsulation host for the AMB-1 magnetotactic bacteria, in order to prevent their motility in presence of an external magnetic field. This mineral matrix can be obtained in water, near neutral pH and at room temperature^35^. It is transparent, chemically-stable and not biodegradable by most living organisms. Such cellular hosts have been previously shown to be compatible with the long-term viability of several strains of bacteria and many other living cells^36^. In these environments, bacteria are confined in a cavity adapted to their size, preventing their motility, and cell division is hindered to insure that the population remains constant over the whole experiment^37^.

In these conditions, it was possible to observe the stable reorientation of magnetosome chains within living magnetotactic bacteria exposed to an external magnetic field. The detailed study of the resulting crystallographic and magnetic structures pointed out the dynamic character of the protein set involved in the organization of the magnetosome chains.

## Results

### Bacterial encapsulation and viability

**Figure 1:**
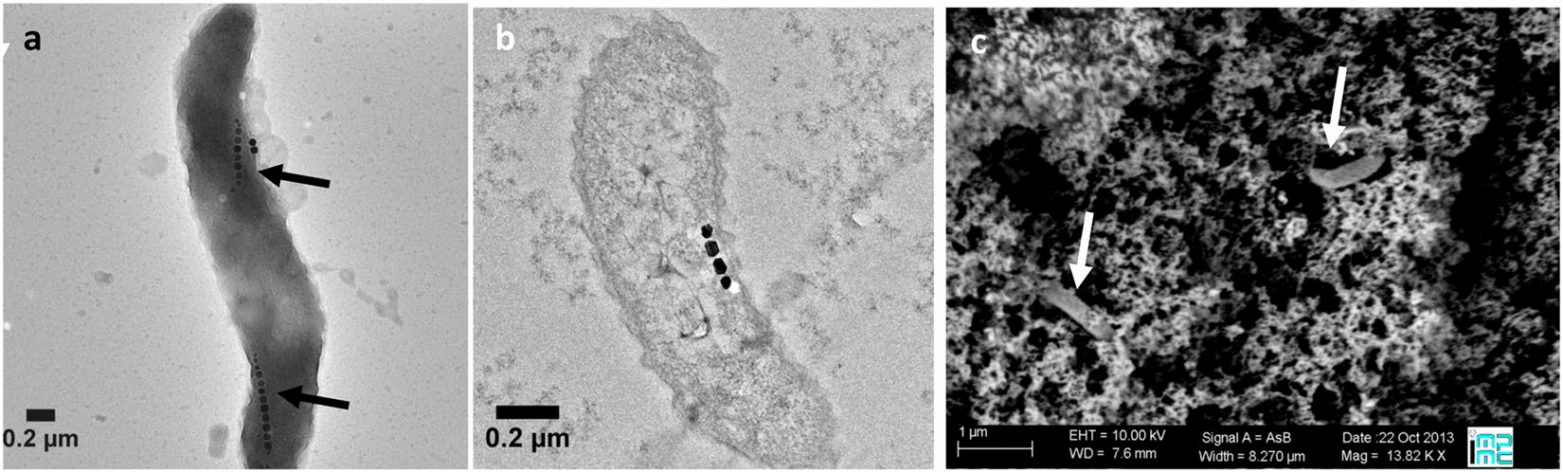
Electron microscopy imaging of free and silica-encapsulated magnetotactic bacteria. (**a**) TEM observation of *M. magneticum* AMB-1 in the initial bacterial suspension (magnetosome chains are indicated by black arrows). Bacteria encapsulated in silica gel observed by (**b**) TEM after ultrasectionning and (**c**) SEM using backscattered electrons after 1 day of encapsulation (bacteria are indicated by white arrows in silica gel).

Initially, AMB-1 cells were cultivated in presence of 20 µM of ferric quinate allowing for the production of magnetosomes. According to the transmission electron microscopy (TEM) observations, these bacteria had as expected biogenic nanoparticles aligned into one or more chains (Fig. 1a). After one day of encapsulation of a bacterial suspension (in phosphate buffer 10 wt% glycerol) in an aqueous silica gel, the ultrastructure of the bacteria remained well preserved (Fig. 1b) and scanning electron microscopy (SEM) images showed that the cells were well dispersed in the porous mineral network (Fig. 1c). Within the encapsulated bacterium in Fig. 1b, magnetosomes with a size typical of 40–50 nm were aligned in chain located on average at 34 nm away from the cytoplasmic membrane. The distance between individual nanoparticles was *ca*. 7 nm. These values were typical of this type of cells, suggesting that the encapsulation process did not impact magnetosome organization^11,12^.

In order to evaluate the viability of the magnetotactic bacteria encapsulated in gels, a colorimetric Alamar Blue test was used to directly quantify the whole viable cells comprising also non-cultivable bacteria. This test allows for the monitoring of the oxygen-consuming metabolism of the cells and was shown to fully correlate with the plate count technique for bacterial viability quantification in the case of silica-encapsulated bacteria^38^. The Alamar Blue test indicated that after 7 days of encapsulation, 28% (+/−4%) of bacteria were alive in gels (Fig. S1). In parallel, the bacterial gels were exposed to an external static magnetic field of 80 mT by means of two parallel magnets placed on either side of flasks containing cells (Fig. S2). A nearly identical viability rate of 27% (+/−4%) was obtained after 7 days, indicating that the magnetic field did not significantly affect the viability of the bacteria over this period (Fig. S1).

### Magnetic properties of bacteria encapsulated in silica gel

**Figure 2:**
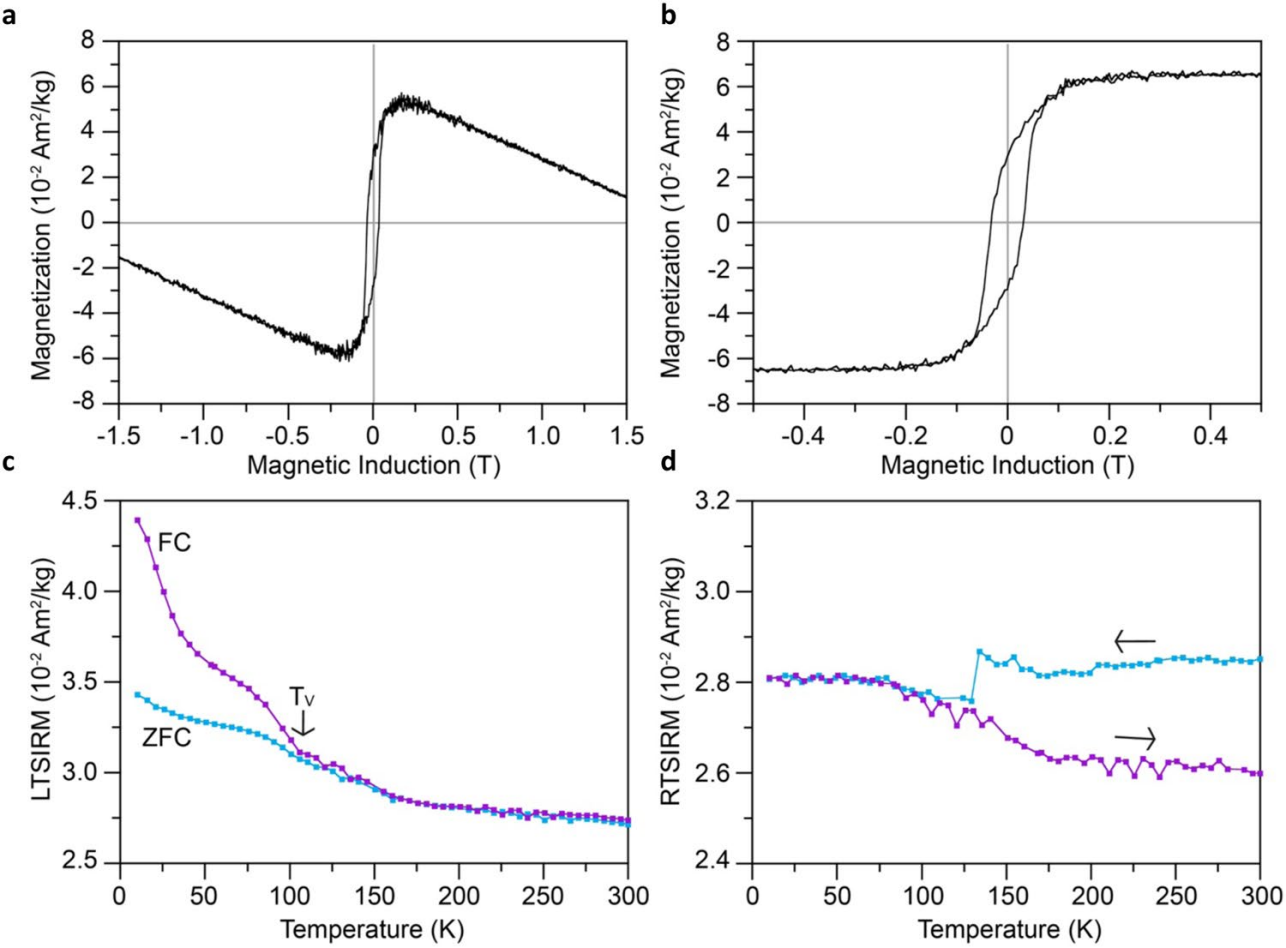
Magnetic properties of silica-encapsulated magnetotactic bacteria. Magnetization curves for the AMB-1 cell-containing aerogel after 1 day acquired at 300 K (**a**) between −1.5 T and +1.5 T and (**b**) between −0.5 T and +0.5 T with correction of the diamagnetic component. (**c**) Field Cooling (FC) and Zero Field Cooling (ZFC) thermal demagnetization curves of the low-temperature saturation isothermal remanent magnetization (LT-SIRM) acquired at 10 K in a 2.5 T induction for the AMB-1 bacterial aerogel after 1 day. (**d**) Cooling (in blue) and warming (in purple) curves of the room-temperature saturation isothermal remanent magnetization (RT-SIRM) acquired at 300 K in a 2.5 T magnetic induction.

Direct current (DC) induced magnetizations and backfield demagnetizations of the bacteria encapsulated in silica aerogel after 1 day were acquired at room temperature. The hysteresis loop displayed a magnetic behavior typical of a mixture between diamagnetic (negative slope of the curve, noticeable at high fields) and ferro- or ferri-magnetic (open hysteresis) materials (Fig. 2a). Mathematical subtraction of the diamagnetic contribution allowed us to isolate the ferrimagnetic contribution of the nanocrystals (Fig. 2b). The sample was characterized by saturation magnetization (Ms) and saturation remanent magnetization (Mrs) values of 0.065 and 0.030 Am2.kg-1, respectively. The saturation magnetization was very low due to the dispersion of the cells within the silica matrix. The sample coercivity (Bc) and coercivity of remanence (Bcr) obtained from backfield measurements were 32 and 38 mT, respectively. The Bcr/Bc and Mrs/Ms ratios were 1.19 and 0.46, respectively. These values are close to those previously reported for whole cell AMB-1^39–42^.

Thermal demagnetization curves of low-temperature saturation remanent magnetization (acquired in 2.5 T at 10 K) after cooling from room temperature in zero magnetic field (zero field cooling, ZFC) or in a 2.5 T magnetic induction (field cooling, FC) displayed a drop in magnetization around 100–110 K characteristic of the Verwey transition, in agreement with previous other AMB-1 samples^39^ (Fig. 2c). The Verwey transition was also clearly visible on the cooling and warming curves of the room temperature (acquired at 300 K in 2.5 T), attesting that bacterial nanocrystals corresponded to magnetite (Fig. 2d)^43^. In addition, the rather flat aspect of the cooling curve above the transition indicated that our samples were not oxidized. *δ*_*FC*_ and *δ*_*ZFC*_ ratios^41^ were equal to 0.14 and 0.09, respectively. The *δ*_*FC*_*/δ*_*ZFC*_ ratio of 1.5 was below the commonly accepted cut-off value of 2 for intact chains of magnetosomes. This suggests that: (i) some chains were disrupted during the CO_2_ supercritical drying for aerogel preparation or (ii) bacteria were encapsulated at different stages of magnetosome chains formation^39,41,44^.

### Effect of the magnetic field on magnetosome orientation

**Figure 3:**
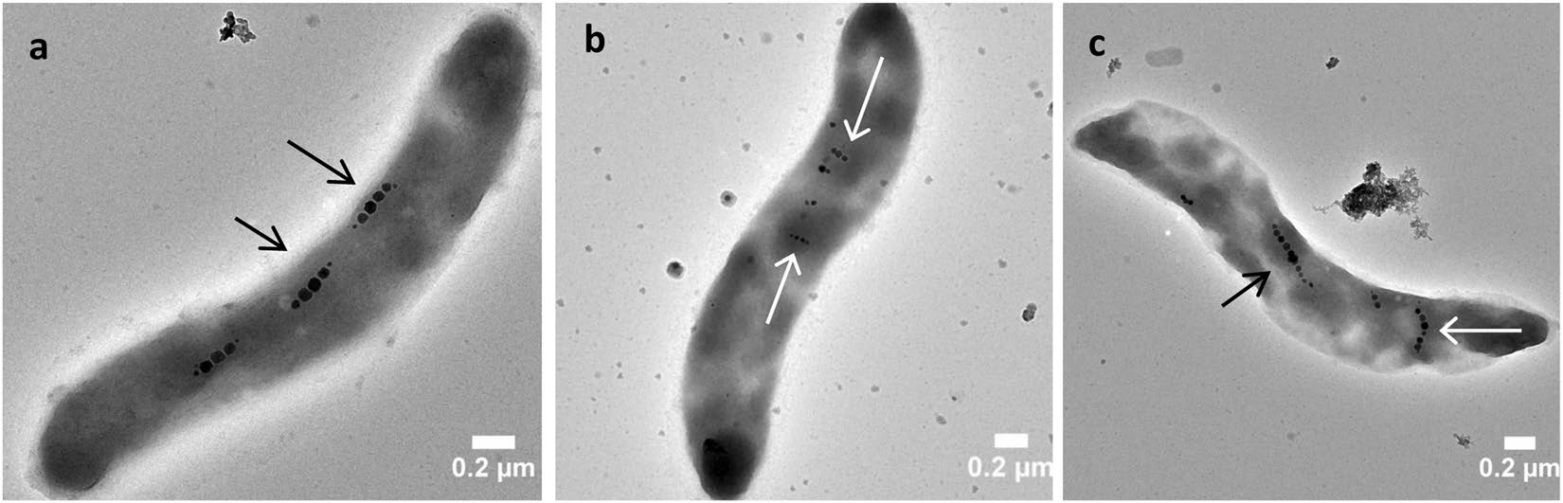
TEM images of silica-encapsulated magnetotactic bacteria exposed, or not, to an external magnetic field. AMB-1 cells kept in (**a**) absence or (**b**,**c**) presence of an 80 mT magnetic field during 7 days of encapsulation. (Black arrows show undeviated chains and white arrows show deviated chains).

The bacteria entrapped in silica matrix without further iron supply (to limit the production of new magnetosomes) were observed after 7 days of exposition or not to an external magnetic field of up to 80 mT (Fig. 3). Whereas the orientation of the magnetosome chains parallel to the bacterial longitudinal axis was preserved in the absence of magnetic field (Fig. 3a), some of the bacteria exposed to the 80 mT field presented several chains of magnetosomes disposed parallel between them but inclined relative to the longitudinal axis of the bacteria (Fig. 3b). Noticeably, those two orientations were sometimes observed within one cell (Fig. 3c). The length of magnetosomes chains oriented transversely to the cell body varied from one bacterium to another (chains of 3 to 6 nanocrystals) (Figs 3b,c and S3). Importantly, bacteria in suspension after 7 days in presence of a magnetic field presented the same magnetosome orientations as in the initial bacterial suspension, indicating that these unusual chain orientations were due to the combination of cell immobilization and external magnetic field.

**Figure 4:**
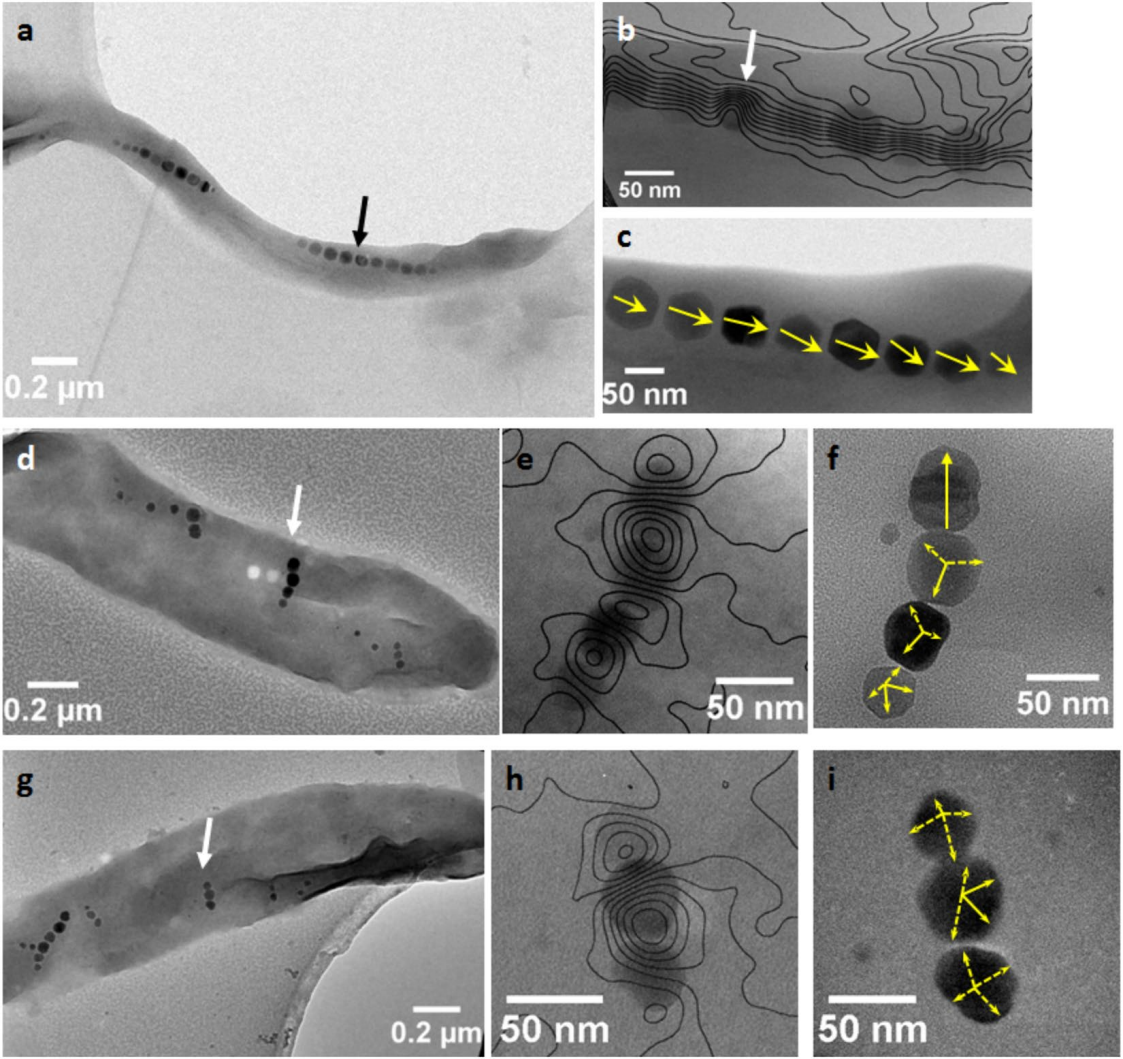
Electron microscopy observations of magnetic and crystallographic orientations in magnetosomes. (**a**) TEM image of an AMB-1 cell in suspension observed after 7 days of incubation in absence of a magnetic field (black arrow shows the selected chain for off-axis image), (**b**) corresponding magnetic phase contours of magnetosome chains determined by off-axis EH (**d**,**g**) TEM images of encapsulated AMB-1 bacteria observed after 7 days of incubation in presence of a magnetic field (white arrows show the selected chains for off-axis images), (**e**,**h**) corresponding magnetic phase contours of magnetosomes chains determined by off-axis EH and (**c**,**f**,**i**) corresponding HRTEM images with 〈111〉 directions determined by using Selected Area Electron Diffraction (SAED) and materialized by yellow bars.

Further information was obtained using off-axis Electron Holography (EH), a powerful interferometric TEM method to correlate morphological microscopy information and local magnetic measurements of nanomaterials^45–49^. This technique allows a quantitative mapping of the magnetic properties of magnetosome chains at the nanoscale^26,50–54^. Here, bacteria with pre-existing magnetosomes in suspension and unexposed to the external magnetic field were first observed after 7 days (Fig. 4a). The contour map of the magnetic flux inside and outside the magnetosome chains suggests that the biogenic particles were coupled parallel and that the magnetization of each magnetosome was aligned forming a magnetic dipole as usually observed in magnetotactic bacteria^26^ (Fig. 4b,c). Encapsulated bacteria were observed after 7 days of exposure to a magnetic field (Fig. 4d,g). In bacterium containing only chains deviated off the cell longitudinal axis (Fig. 4d), off-axis EH images displayed anti-parallel coupling between the magnetosomes (Fig. 4e) whereas for bacterium exhibiting both parallel and deviated chains off the cell longitudinal axis (Fig. 4g), two magnetic configurations were observed: (i) an anti-parallel coupling in deviated chain (Fig. 4h) and (ii) a parallel coupling in parallel chain (Fig. S4).

Complementary experiments were performed to determine how the magnetosomes re-oriented in the applied magnetic field. High-resolution TEM (HRTEM) analyses were used to determine the crystallographic axes in nanoparticles from different chains previously studied by electron holography. In the magnetosome chain of the bacterium not exposed to the magnetic field, the [111] axes were aligned parallel to the bacterial longitudinal axis (Figs 4c and S5), as already observed for *Magnetospirillum* sp.^24,26^. The same crystallographic orientation was observed for the bacterium containing only chains deviated off the longitudinal cell axis (Figs 4f and S6) and for the bacterium where both chain orientations coexisted (Figs 4I and S7). Some [111] axes in deviated chains in both bacteria (Fig. 4f,i) were oriented in the direction or close to the direction of the particles magnetization (Fig. 4e,h). In the chain placed along the longitudinal axis of the cell (Figs 4g, S4 and S8), the [111] axes of magnetosomes preserved their orientations parallel to the magnetization and in the chain direction.

## Discussion

So far, the influence of a magnetic field on magnetosome within magnetotactic bacteria has been mainly studied on cell suspensions, showing noticeable effects on magnetosome size, morphology and rate of production^27–30^. One study by Körnig *et al*. reported the effect of a magnetic field on MSR-1 bacteria entrapped within an agarose hydrogel^34^. At sufficiently high magnetic field (35 mT and above), a rotation of the nanocrystals to align along the field direction was reported. When these cells were fixed with paraformaldehyde (i.e. preserving the intracellular organization when the field is turned off), short chains were observed that were also aligned along the field direction. Such a disruption of the chain organization was further evidenced by application of a second field that could re-orientate the chains only if the first field had an intensity above 35 mT. Interestingly, if the first field was turned off without cell chemical fixation, the initial chains were recovered. Such reversibility would suggest that the assembly of MamK/MamJ proteins responsible for chain integrity had a strong dynamic character, allowing for chain reconstruction after the field-induced disruption.

Compared to this previous study, our system has several specificities. First it must be pointed out that the magnetotactic bacteria strain we used, AMB-1, does not show the exact same set of proteins as MSR-1^16–23^, which could at least explain some of the differences between the two systems. Contrary to some magnetotactic bacteria which are difficult to isolate, cultivate^55^ or are obligate microaerophile^56,57^, AMB-1 bacteria grow in liquid culture medium and on agar plates in presence of oxygen^58^. This strain is not obligate microaerophile and can therefore be easily manipulated for various applications^2,59,60^. The incubation of these oxygen-tolerant bacteria in culture medium bottle without stirring and air exchange at 30 °C permitted to obtain microaerophilic conditions favoring the magnetosomes productions. In our experiments, the oxygen tolerance of AMB-1 bacteria limited the stress of the encapsulation in an air atmosphere. Compared to *M. gryphiswaldense*, *M. magneticum* possess additional proteins (MamK-like and MamJ-like) acting in the chain magnetosomes organization and dynamic of the filament^21,23^. Consequently, the cell response to a magnetic environment may depend on the strain and phenotypic divergences^61^. Second, whereas in the previous study, the MSR-1 cells were initially aligned by a magnetic field before their immobilization in agarose, the AMB-1 cells were here more randomly distributed within the silica gel when it formed and trapped the bacteria. In other words, if the observed magnetosome and/or chain orientation was strictly reflecting the external field direction, all organizations within a single bacterium should be the same, in contradiction with our observations. Finally, and more importantly, the chains deviated off the longitudinal cell axis were here observed when the external magnetic field was turned-off without the need for cell fixation. This calls for the examination of alternative mechanisms.

It seems crucial to first consider magnetosome nanocrystals and chains of magnetosomes independently. Electron microscopy studies on bacteria-containing gels after 1 and 7 days in the absence of external field indicated that the intracellular content of the initial magnetotactic bacteria was well-preserved within the silica network. In this situation, single domain magnetite nanoparticles were arranged in chains oriented parallel to the longitudinal axis of the cell. When the magnetic field was applied on the encapsulated bacteria, we observed that (i) some chains were no longer parallel to the cell longitudinal axis and remained so even if the field is turned-off, (ii) in these chains, the magnetosomes interacted via an anti-parallel magnetic coupling, (iii) they preserved their relative crystallographic co-alignment, (iv) these modifications did not seem to have a significant detrimental effect on cell viability (as compared to encapsulated cells aged without external field).

**Figure 5:**
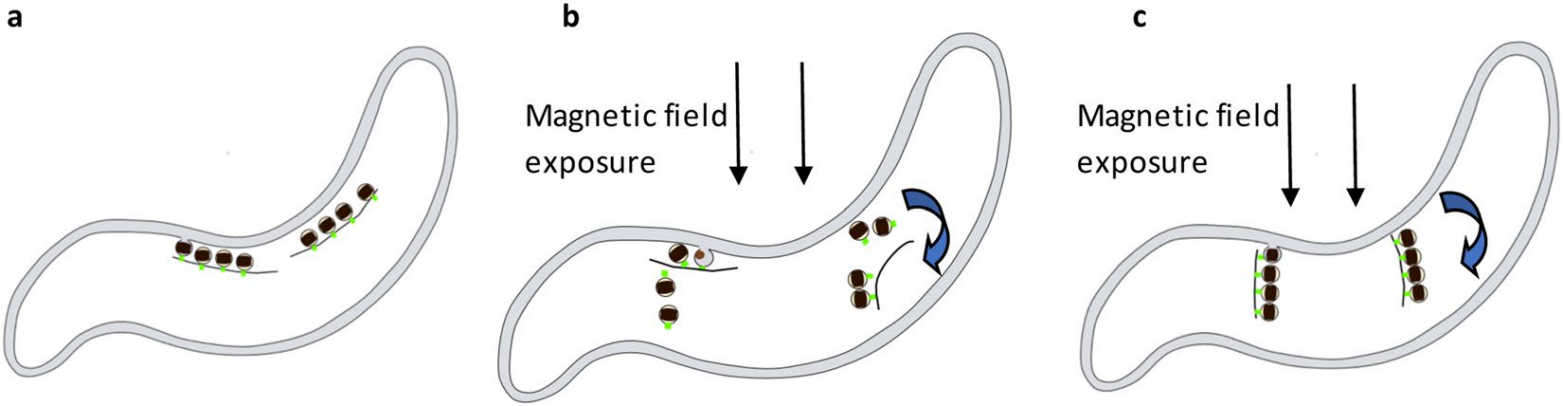
Schematic representation of the possible mechanisms involved in deviated magnetosome chains. (**a**) AMB-1 bacteria with magnetosome chains connected to the cytoplasmic membrane via an invagination and stabilized by the molecular complexe MamK/MamJ. (**b**) Magnetosome chains disrupted during the magnetic field exposition. Each magnetosome responds individually to the field and arranges again in chain thanks to the molecular complex MamK/MamJ. (**c**) Whole magnetosome chains as one entity orient directly in the magnetic field direction.

Some studies of *M. gryphiswaldense* MSR-1, have suggested that the magnetosome vesicles form a distinct compartment detached from the cytoplasmic membrane^17^. The nucleation of magnetite crystals would take place in invagination of the cytoplasmic membrane, favoring iron transfer from the external medium; the vesicles would then detach at some stage of the biomineralization process^62,63^. A dynamic filamentous protein MamK would play a role in the maintenance of chain continuity by reducing gaps between magnetosomes in *M. magneticum* AMB-1^18,64^. Note that some remaining contact between the magnetic chain and the membrane is necessary to permit the alignment of the cell in the geomagnetic field^3^. From this starting situation (Fig. 5a), the effect of the magnetic field can occur according to two hypothetical mechanisms:

(i) The integrity of the whole chain is lost, allowing for the motion of individual magnetosomes in the magnetic field, that could be then collected and reassembled by the dynamic structural proteins MamK and MamJ (Fig. 5b). It has been suggested that the formation of magnetosome chains was a step-by-step process in which each particle is synthesized consecutively so that the orientation of the growing one is imposed by those already present^6,33,65,66^. Here, as each magnetite nanocrystal would respond individually to the magnetic field before being reassembled, their relative crystallographic and magnetic orientation is difficult to predict.
(ii) The structures connecting magnetosomes to the cytoplasmic membrane would be disrupted to the exception of some possible anchoring sites and the protein filament insuring the chain integrity would be preserved. Then the whole chain would turn to align its global magnetic dipole along the external field (Fig. 5c). In this situation the crystalline orientation of the magnetosomes should be preserved whereas modification in the magnetic interactions can occur.

Here, we found out that the crystalline orientation within deviated chains after the field is turned off can be similar to non-deviated chains (i.e. [111] axis parallel to the chain direction). It is important to point out that compared to Körnig *et al*.^34^, the cells were still alive (not chemically fixed) when the field was turned-off so that they could preserve their ability to synthesize the proteins involved in chains organization, orientation and interaction with the inner membrane. Turning to magnetic interactions, anti-parallel coupling has been observed in deviated chains of magnetosomes whereas parallel coupling was present in non-deviated ones. Overall, the magnetic field exposure has favored the destructuration of magnetosome chains organization.

In conclusion, our results shade new light on the mechanisms by which magnetotactic bacteria are able to control the assembly of magnetic nanocrystals within magnetosome chains. Our observation of a stable deviation of the chain orientation within living cells suggests that the assembly of cytoskeletal proteins regulating the nanocrystal organization would have a significant dynamic character, echoing recent evidences of the role of actin in iron oxide biomineralization in honeybees^67^. Some other proteins could also have a role in maintaining stable deviated chains by interacting with magnetosomes and the cytoplasmic membrane. Of significance for the field of magnetotactic bacteria is the fact that the underlying process, and therefore the associated set of proteins, may be specific to AMB-1, providing an additional evidence of phenotypic divergence in this group of biomineralizing bacteria. The here-demonstrated possibility to manipulate chain orientation without impacting cell viability may also open opportunities for the use of encapsulated magnetotactic bacteria within functional devices.

## Methods

### Bacteria culture

*Magnetospirillum magneticum* strain AMB-1 ATCC 700264 was grown in culture medium containing KH_2_PO_4_ (0.68 g/L), Succinic acid (0.37 g/L), Tartaric acid (0.37 g/L), Sodium acetate (0.05 g/L), NaNO_3_ (0.12 g/L), Ascorbic acid (0.035 g/L), *Wolfe’s Vitamin Solution* (10 mL), *Wolfe’s Mineral Solution* (5 mL), and ferric quinate (0.01 M) (2 mL). *Wolfe’s Vitamin Solution* (ATCC) contains Biotin (2 mg/L), Folic acid (2 mg/L), Pyridoxine (10 mg/L), Thiamine (5 mg/L), Riboflavin (5 mg/L), nicotinic acid (5 mg/L), panthotenic acid (5 mg/L), vitamin B12 (0.1 mg/L), p-Aminobenzoic acid (5 mg/L), Thioctic acid (5 mg/L). *Wolfe’s Mineral Solution* (non ATCC) contains Nitrilotriacetic acid (0.5 g/L), MgSO_4_.7H_2_O (1.5 g/L), MnSO_4_.H_2_O (1 g/L), NaCl (0.5 g/L), FeSO_4_. 7 H_2_O (0.1 g/L), CoNO_3_. 6 H_2_O (0.1 g/L), CaCl_2_ (0.1 g/L), ZnSO_4_.7 H_2_O (0.1 g/L), CuSO_4_.5 H_2_O (0.01 g/L), AlK(SO_4_)_2_.12 H_2_O (0.01 g/L), H_3_BO_3_ (0.01 g/L), Na_2_MoO_4_ (0.01 g/L), Na_2_SeO_3_ (0.002 g/L), EDTA (0.5 g/L). The pH of the medium was adjusted to 6.75 with sodium hydroxide solution (1 M) and sterilized.

1.8 mL of AMB-1 strain culture, stored at −80 °C, are inoculated in a flask of 20 mL with 15 mL of culture medium containing ferric quinate (20 µM). The bacteria preculture is incubated at 30 °C for 4 to 10 days. 10 mL of preculture are then added in a bottle of 1 L containing 1 L of iron-rich culture medium (20 µM of ferric quinate) to permit the production of magnetosomes. The incubation is performed over 7 days at 30 °C.

### Bacteria encapsulation

The culture with magnetosomes was centrifuged at 6000 × g during 30 minutes at 5 °C, then the pellet was suspended with 4 mL of phosphate buffer (pH 7.2) containing 10 wt% glycerol. The bacterial suspension was adjusted to an optical density (OD) of 5. Silica gels was prepared from a precursor of sodium silicate solution (27 wt% SiO_2_, 10 wt% NaOH) from Sigma Aldrich. A mixture of sodium silicate (0.8 M, 1 mL) and glycerol (10 wt%, 1 mL) was neutralized with hydrochloric acid (4 M, 0.155 mL) under stirring. After 5 seconds, 1 mL of bacterial suspension (OD = 5) was added and the assemblage was stirred for 5 minutes. The gels were formed after 5–6 min at room temperature and were then kept at 20 °C for up to 7 days.

The encapsulated bacteria were exposed to an external static magnetic field created by two parallel magnets. The magnets were placed in a set-up realized with Plexiglas^TM^ and were spaced of 5 cm that permitted to have a field of 55 mT in the center of the set-up and 80 mT at each magnet (see Fig. 2b).

### Characterization of bacteria-silica aerogels

In order to obtain dried material, AMB-1 containing silica gels were converted into aerogels via supercritical drying as described thereafter. Shortly, the bacterial gels are first fixed with a glutaraldehyde solution (2.5%) for 1 hour and washed 3 times in a buffer. Then the samples are dehydrated in different baths of ethanol and dried by using a CO_2_ supercritical point dryer.

The aerogel magnetization was first measured as a function of the applied field magnetic intensity at 300 K using a Vibrating Sample Magnetometer (VSM) model 3900 from Princeton Measurements Corporation. Low-temperature measurements were performed using a Superconducting Quantum Interferference Device (SQUID) magnetometer MPMS XL-5 from Quantum Design.

Scanning electron microscopy (SEM) was performed using a Zeiss Ultra 55 SEM-FEG microscope on a carbon-coated aerogel crushed sample.

### Characterization of silica-extracted bacteria

Bacterial gels were dispersed in 5 ml of phosphate buffer, then broken with 4 mm glass beads under stirring for 15 minutes. The resuspended gels were then diluted in phosphate buffer before performed viability tests using the Alamar Blue method^38^.

For TEM observations, the resuspended gels were centrifuged at 600 × g at 10 °C during 15 min to sediment the gel aggregates. The supernatant containing bacteria was collected and diluted in phosphate buffer. All diluted suspensions were washed by centrifugation at 6000 × g at 10 °C during 15 min. The supernatant was removed and 3 ml of the bottom were kept and homogenized. 10 µL of concentrated bacterial suspension were placed on copper grids covered with thin carbon film and dried at 30 °C during 3 hours to permit sedimentation of cells on the carbon film. Then the bacteria were fixed with 10 µL of glutaraldehyde (2.5%) for 1 hour at 4 °C. The samples were then washed with ultrapure water and dried before the observation by TEM using a JEOL TEM 2100 equipped with an LaB6 crystal gun, with an accelerating tension of 200 kV. The high-resolution TEM (HRTEM) has been performed on the same samples by using a TEM JEOL-2100F operating at 200 kV and equipped with a field emission gun (FEG). The in-plane crystallographic direction and observed zone axes have been determined by using the Fast Fourier transform on the two-dimensional HRTEM images and have permitted to deduce then the corresponding stereographic projection for each particle. Off-axis electron holography (EH) was carried out using Hitachi HF3300C microscope, following previously described protocols^26,45,46,68,69^. Ultrathin sections of bacterial gels were prepared according to the following protocol. The bacterial gels were first fixed with a glutaraldehyde solution (2.5%) for 1 hour and washed 3 times in a buffer, then fixed with osmium tetroxide solution (2%) for 1 hour before washed 3 times in a buffer. The samples were then dehydrated in different baths of ethanol and embedded in Araldite ® resin.

### Data availability

No datasets were generated or analysed during the current study.

## Electronic supplementary material

supplementary information
