# Supplementary material for: Magnetic-field induced rotation of magnetosome chains in silicified magnetotactic bacteria
Source: Sci Rep. 2018 May 16;8:7699. doi: 10.1038/s41598-018-25972-x (PMC5955880; doi:10.1038/s41598-018-25972-x)
Supplement: Supplementary file 1 — supplementary information [file 41598_2018_25972_MOESM1_ESM.pdf]

Supplementary Information

Magnetic-field induced rotation of magnetosome chains in silicified magnetotactic bacteria

Marine Blondeau, Yohan Guyodo, François Guyot, Christophe Gatel, Nicolas Menguy, Imène Chebbi, Bernard Haye, Mickaël Durand-Dubief, Edouard Alphandery, Roberta Brayner, & Thibaud Coradin

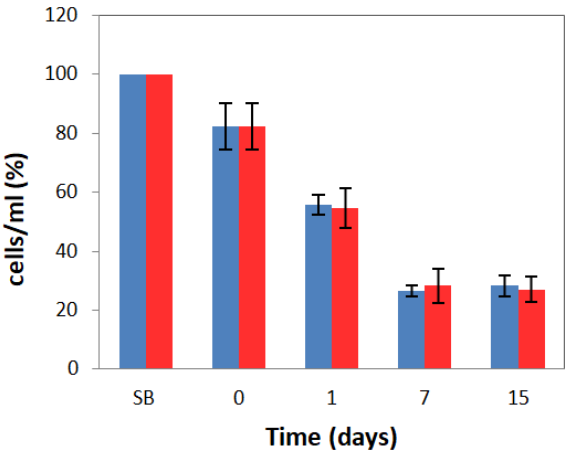

Figure S1. Viability of silica-encapsulated magnetotactic bacteria in the presence (red) or absence (blue) of an external magnetic field, as determined by the Alamar Blue assay

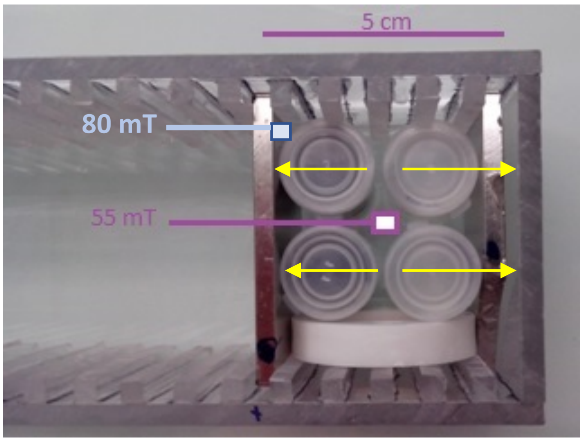

Figure S2. Experimental set-up for application of an external magnetic field. The samples were placed between two similar magnetic plates to obtain a magnetic field oriented from the center (55 mT) to the plates (80 mT) (direction indicated by yellow arrows)

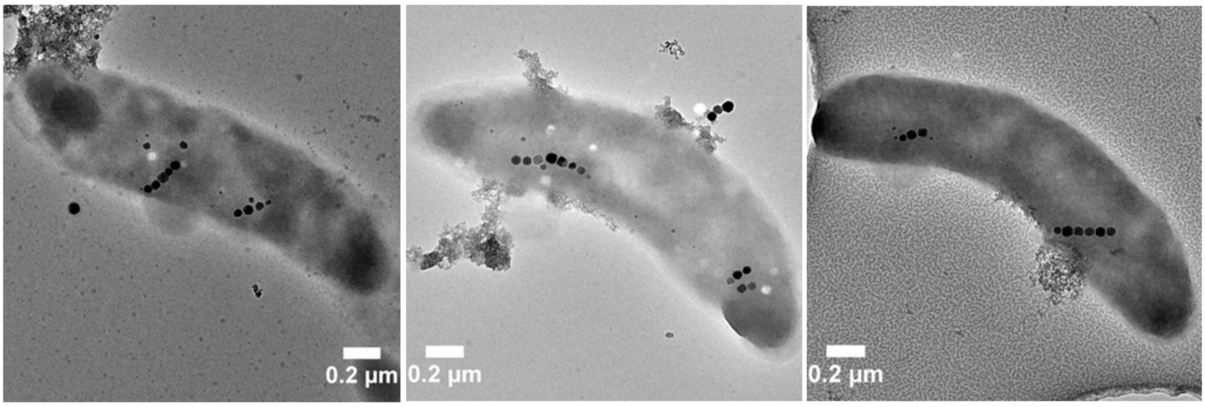

Figure S3. TEM images of silica-encapsulated magnetotactic bacteria exposed to an external magnetic field during 7 days of encapsulation

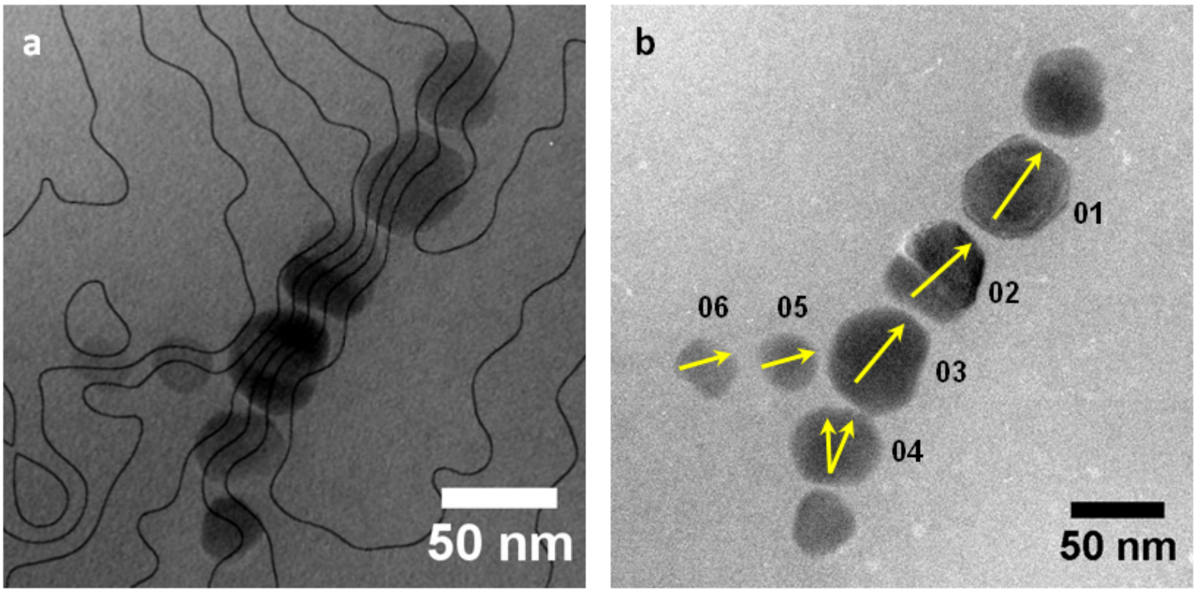

Figure S4. Electron microscopy studies of magnetic and crystallographic orientations in magnetosomes. (**a**) Magnetic phase contours of no deviated magnetosomes chain in Fig.4g determined by off-axis EH. (**b**) HRTEM images on magnetosomes chain with <111> directions materialized by yellow bars.

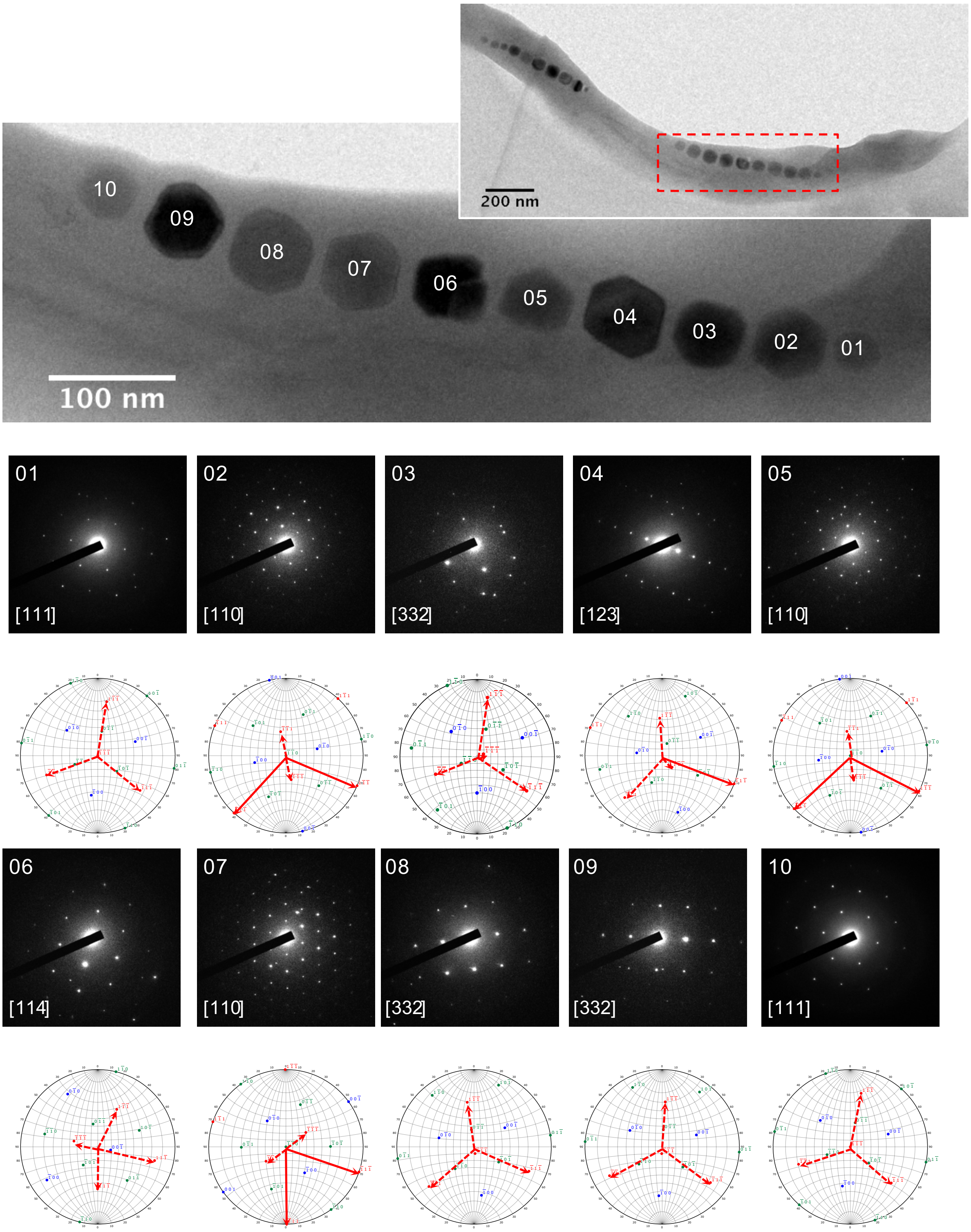
200 nm
100 nm
10
09
08
07
06
05
04
03
02
01
01 [111]
02 [110]
03 [332]
04 [123]
05 [110]
06 [114]
07 [110]
08 [332]
09 [332]
10 [111]

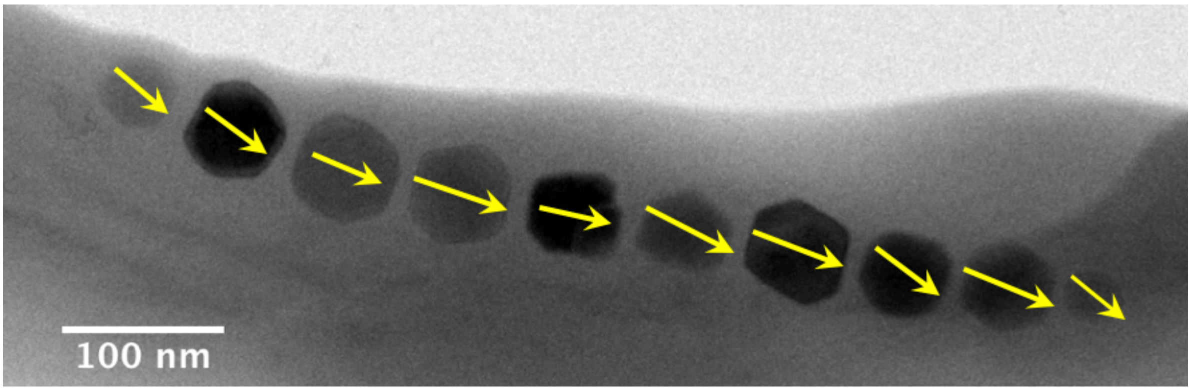

Figure S5. HRTEM images on magnetosomes of the chain in Fig. 4c. <111> directions determined by using Selected Area Electron Diffraction (SAED).

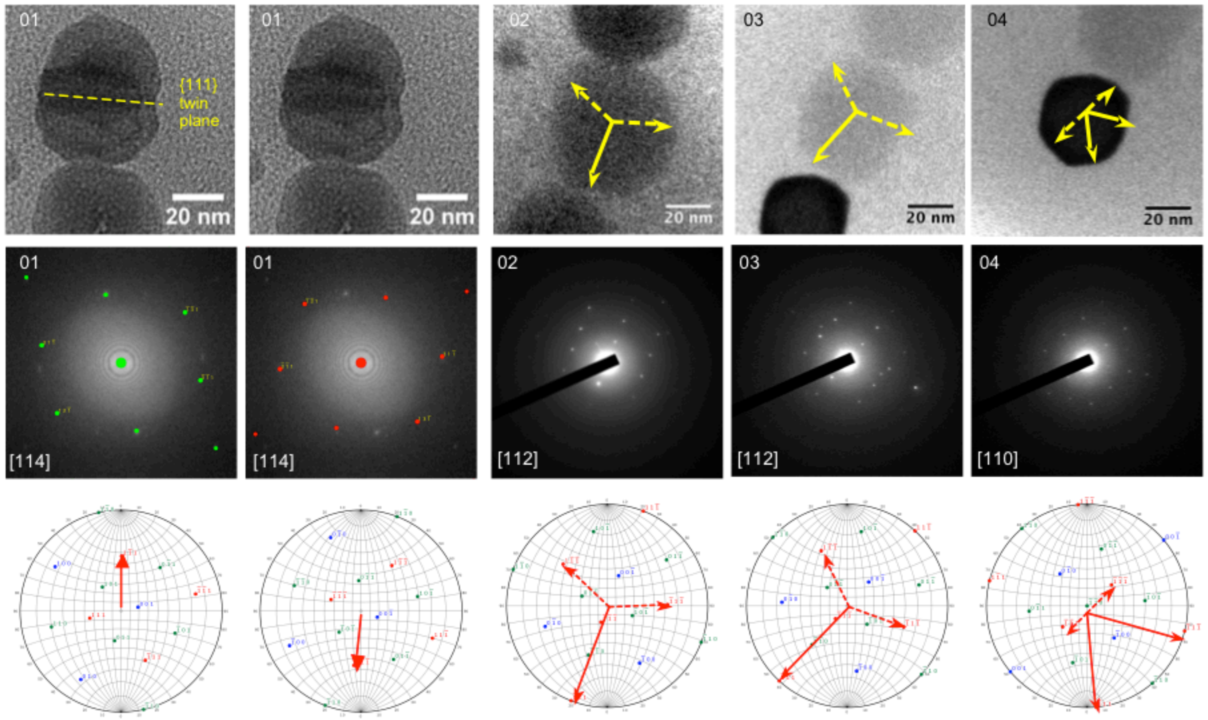

Figure S6 : HRTEM images on magnetosomes of the chain in Fig. 4f. <111> crystallographic directions determined by using Selected Area Electron Diffraction (SAED) or Fast Fourier Transform (FFT) or HRTEM images.

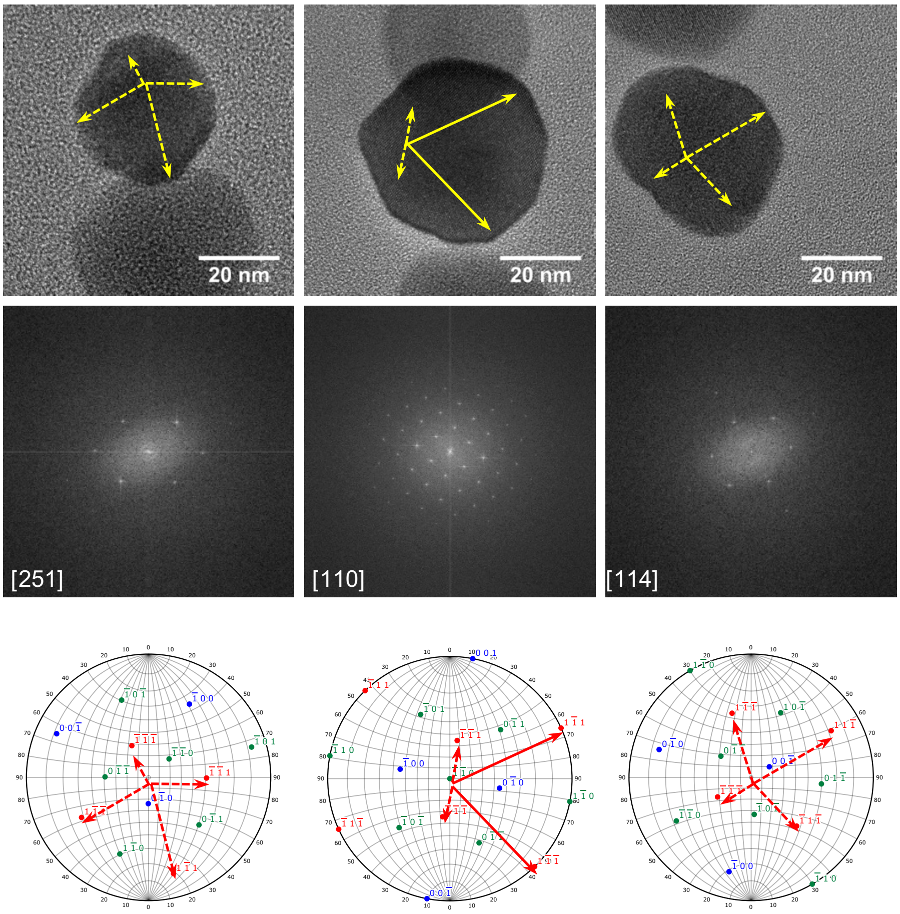

Figure S7 : HRTEM images on magnetosomes of the chain in Fig. 4i. <111> directions determined from Fast Fourier Transform (FFT) on HRTEM images.

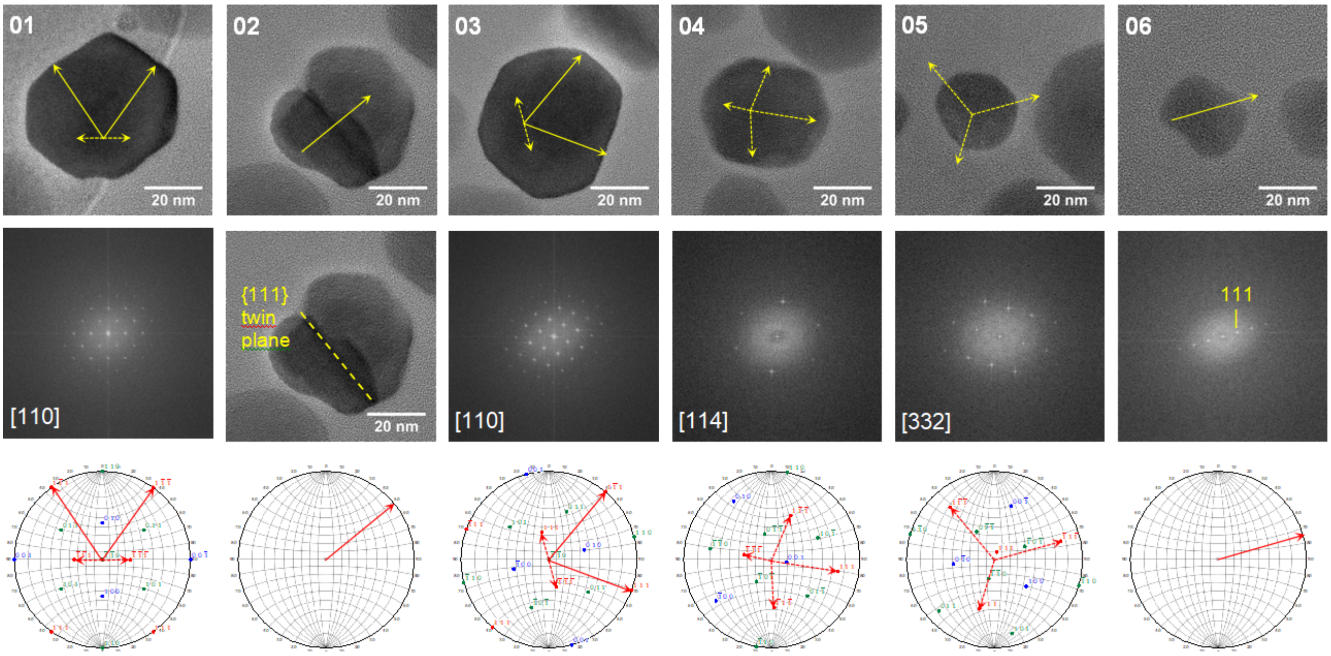

Figure S8. HRTEM images on magnetosomes of the chain in Fig. S4. <111> crystallographic directions determined from Fast Fourier Transform (FFT) on HRTEM images or trace of {111} twin boundary.
